# Comparison of normalization methods for CodeLink Bioarray data

**DOI:** 10.1186/1471-2105-6-309

**Published:** 2005-12-28

**Authors:** Wei Wu, Nilesh Dave, George C Tseng, Thomas Richards, Eric P Xing, Naftali Kaminski

**Affiliations:** 1 Dorothy P. and Richard P. Simmons Center for Interstitial Lung Disease, Division of Pulmonary, Allergy and Critical Care Medicine, University of Pittsburgh Medical Center, Pittsburgh, PA 15213, USA; 2 Department of Biostatistics, University of Pittsburgh, Pittsburgh, PA 15261, USA; 3 Center for Automated Learning and Discovery and Language Technology Institute, School of Computer Science, Carnegie Mellon University, Pittsburgh, PA 15213, USA

## Abstract

**Background:**

The quality of microarray data can seriously affect the accuracy of downstream analyses. In order to reduce variability and enhance signal reproducibility in these data, many normalization methods have been proposed and evaluated, most of which are for data obtained from cDNA microarrays and Affymetrix GeneChips. CodeLink Bioarrays are a newly emerged, single-color oligonucleotide microarray platform. To date, there are no reported studies that evaluate normalization methods for CodeLink Bioarrays.

**Results:**

We compared five existing normalization approaches, in terms of both noise reduction and signal retention: Median (suggested by the manufacturer), CyclicLoess, Quantile, Iset, and Qspline. These methods were applied to two real datasets (a time course dataset and a lung disease-related dataset) generated by CodeLink Bioarrays and were assessed using multiple statistical significance tests. Compared to Median, CyclicLoess and Qspline exhibit a significant and the most consistent improvement in reduction of variability and retention of signal. CyclicLoess appears to retain more signal than Qspline. Quantile reduces more variability than Median in both datasets, yet fails to consistently retain more signal in the time course dataset. Iset does not improve over Median in either noise reduction or signal enhancement in the time course dataset.

**Conclusion:**

Median is insufficient either to reduce variability or to retain signal effectively for CodeLink Bioarray data. CyclicLoess is a more suitable approach for normalizing these data. CyclicLoess also seems to be the most effective method among the five different normalization strategies examined.

## Background

DNA microarrays have made possible the expression profiling of thousands of genes in a single experiment. They have been used in a wide range of applications, *e.g*., disease classification and characterization [1-4], discovery of new disease subtypes [5,6], and identification of novel genes or gene regulatory networks for biological mechanistic research [7,8]. Depending on the probe types used, DNA microarrays can be classified as either cDNA or oligonucleotide microarrays. Additionally, depending on the number of samples hybridized to a single array, microarrays can be classified as single-color or two-color arrays. CodeLink Bioarrays are recently developed, single-color oligonucleotide microarrays, which differ from Affymetrix GeneChips (the largest and most established microarray manufacturer) mainly in that, in order to detect a target transcript, CodeLink Bioarrays use a single 30-mer oligonucleotide probe, whereas GeneChips use multiple 25-mer probes. CodeLink Bioarrays have been applied successfully to identify and characterize target genes involved in idiopathic pulmonary fibrosis [9] and emphysema [10], to identify molecular signatures in colon cancer development [11] and postnatal uterine development [12], and to elucidate transcriptional mechanisms underlying the osteogenic process [13].

Regardless of platforms, microarray data are noisy due to the co-existence of genuine biological variations (signal) and noise. Signal is desirable and makes samples of different biological natures distinguishable from one another. Noise, however, is of no biological relevance and can arise from any step: sample preparation, labelling, hybridization, or scanning [14]. Moreover, noise can hide meaningful biological signal and seriously skew the results of downstream data analyses. Therefore, it must be minimized to ensure the accuracy of downstream studies. In order to do so, many normalization strategies have been proposed and evaluated, but most have been for data obtained from cDNA microarrays [15,16] and from Affymetrix GeneChips [17,18]. Since both CodeLink Bioarrays and GeneChips are single-color oligonucleotide microarrays, data generated from these platforms can usually be normalized by the same approaches. However, because of differences in array designs, methods that perform well for one platform may not work equally well for the other. It is, therefore, still necessary to validate normalization methods for CodeLink Bioarrays. To date, there have been no reported studies that evaluated normalization methods for CodeLink arrays.

A challenge inherent for the normalization of microarray data is the lack of a gold standard (*e.g*., a 'perfect' validation set that can reflect the complexity of real data). It is, therefore, not easy to identify the best normalization method(s) (which can both remove noise and retain signal most effectively) for any array platform. Two criteria have been employed to compare normalization strategies [17-19]: one is the ability to reduce noise in the data and the other is the ability to retain biological signal. To assess noise reduction, diagnostic plots have been employed to visualize and identify noise in the data. For example, the MA plot, which is the scatter plot of average log intensity values (*A*) of the green and red fluorescent dyes (in two-color cDNA microarrays) vs. log intensity ratio (*M*) of the two fluorescent dyes, has been used to identify intensity-dependent dye effect in cDNA microarray data [19]; while the spatial plot, a spatial representation of the spots on a microarray color-coded by their intensity values, has been used to evaluate spatial-dependent dye effect in cDNA microarray data [20]. Other approaches have used the coefficient of variation [21,22], correlation [21] and variance [23] in replicate arrays, as quantitative, unambiguous measures to evaluate the effectiveness of normalization methods in removing variability.

It is relatively ambiguous to evaluate normalization methods in terms of signal retention, because there is no ground truth for total signal in real biological samples. Prevailing approaches compare the ability to predict a fixed number of known differentially expressed genes using *t*-statistics [19] and adjusted *p*-values [24] and to reveal spike-in genes [17]. When little is known about differentially expressed genes in the data (a common situation when real datasets are used), signal has been estimated by calculating the overabundance of differentially expressed genes or informative genes from real datasets [18]. The leave-one-out cross-validation *k*-NN classification error has also been employed as a functional measure for comparing normalization methods in real datasets [16].

Previously we have developed a strategy for the evaluation of normalization approaches for Affymetrix GeneChips [18]. In the present study, we employed a similar strategy to compare five existing normalization methods (Median, CyclicLoess, Quantile, Iset, and Qspline) for CodeLink Bioarray data. These methods were designed for high-density oligonucleotide microarrays, and some of them (Median, CyclicLoess, Quantile, and Iset) have already been validated using GeneChip data [17,18]. We applied these normalization approaches individually to two real datasets obtained from CodeLink Bioarrays. We examined intensity-dependent differences in the normalized datasets and assessed variability of the normalized datasets, using replicate microarrays and/or positive control probes on the arrays. Finally, to assess the effectiveness of the normalization methods in retaining signal, we compared the numbers of differentially expressed genes detected from the normalized datasets using multiple statistical significance tests. Computer programs in this work were implemented using the R language for statistical computing and graphics [25].

## Results

### Noise in the normalized time course dataset

#### Intensity-dependent differences in the normalized data

**Figure 1:**
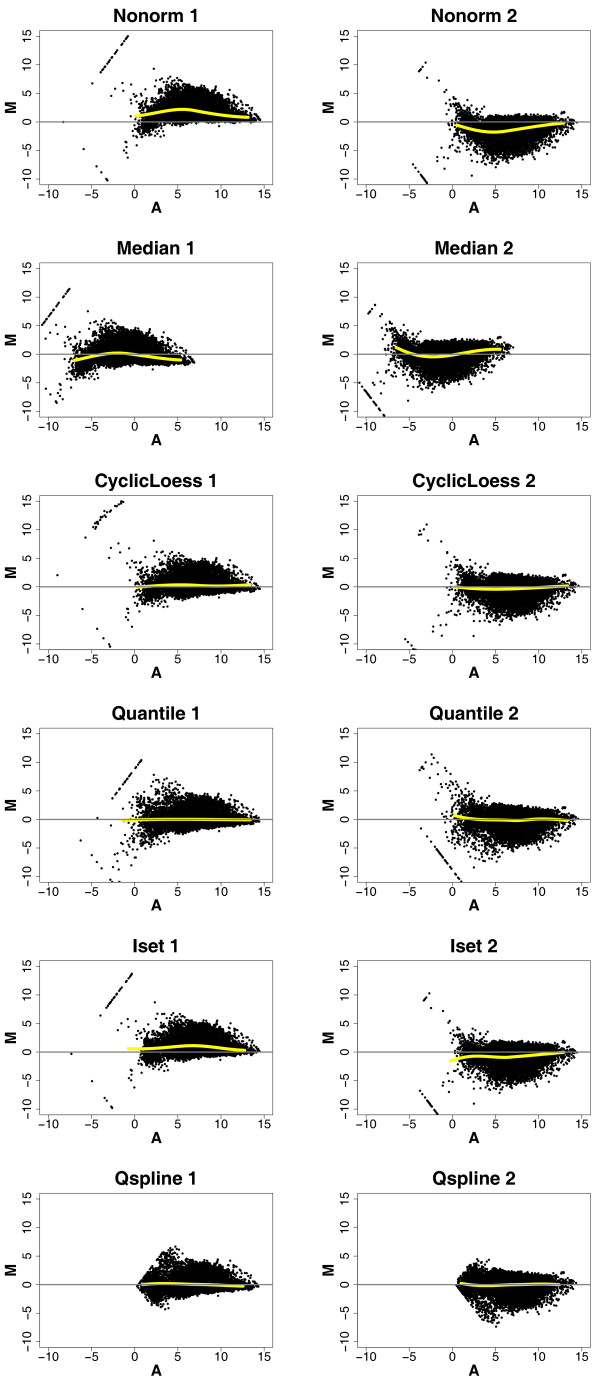
**MA plots of two pairs of microarrays in the TRC1 replicate set from the time course dataset**. The plots in each row show the results from non-normalized data (row 1) and data normalized with each of the five normalization methods (row 2–6). Columns depict two pairs of microarrays, pair 1 (array 1 vs. array 4, left) and pair 2 (array 4 vs. array 5, right), in the TRC1 replicates that exhibit obvious intensity-dependent differences between the arrays. The yellow line in each plot shows the loess fitting of the entire data in the plot.

We used pairwise MA plots to examine the ability of the normalization methods to remove intensity-dependent differences between each pair of arrays in the technical replicates. Figure 1 shows the MA plots of two pairs of arrays in the TRC1 replicate data, either non-normalized (Nonorm), or normalized with the five normalization methods. These arrays exhibit obvious intensity-dependent differences between "pair 1" (array 1 vs. array 4) and "pair 2" (array 4 vs. array 5) (shown as curved loess lines in the plots) (Figure 1, Nonorm). For the Median-normalized data, although the loess lines are centered near zero, they still have curvature, indicating that Median does not remove intensity-dependent differences adequately (Figure 1, Median). CyclicLoess, Quantile and Qspline remove intensity-dependent differences almost completely; Iset, however, fails to remove all the intensity-dependent differences (Figure 1).

#### Variability of the normalized data

**Figure 2:**
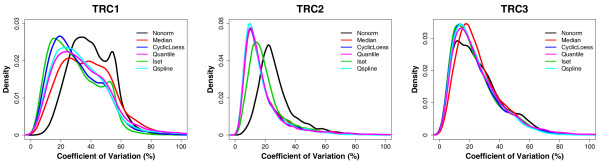
**Comparison of variability of the normalized data in the three sets of technical replicates from the time course dataset**. The density plots show the CVs of all data points (either non-normalized or normalized with the five normalization methods) from each set of the technical replicates (TRC1, TRC2 and TRC3).

We calculated the coefficient of variation (CV) of the normalized intensity values for each transcript across all arrays in each set of the normalized technical replicates. Figure 2 shows the density plots of the CVs for Nonorm and the five normalization methods. The CV of Nonorm in TRC1 peaks more to the left side with a wider spread than those in TRC2 and TRC3, suggesting that the quality of the TRC1 replicates is not as good as those of TRC2 and TRC3. This is consistent with our observation that TRC1 contains two 'corrupted' pairs of arrays that show obvious intensity-dependent differences in the MA plots (Figure 1). Figure 2 shows that in TRC1, the five normalization methods reduce the data variability to different degrees: Iset and CyclicLoess have overall lower CVs (and thus perform better) than Median, Quantile and Qspline, as indicated by the CV density plots of the former methods peak more to the left side of those of the latter approaches; Quantile and Qspline have slightly lower CVs than Median. In TRC2 (Figure 2), Iset fails to improve over Median, whereas the performances of the other four normalization methods (CyclicLoess, Quantile, Qspline, and Median) are similar. In TRC3, Median is outperformed by all the other four normalization strategies (Figure 2).

Finally, we calculated the CVs of the normalized intensity values for positive control probes across all arrays in the time course dataset. Our results show that CyclicLoess, Quantile and Qspline reduce variability from the normalized data more effectively than Nonorm, Median and Iset. The means of the CVs for CyclicLoess, Quantile and Qspline are 57%, 54% and 56% respectively, whereas the means of the CVs for Nonorm, Median and Iset are 72%, 66% and 61% respectively. CyclicLoess has a slightly higher mean CV than Quantile and Qspline, but the differences are not statistically significant (Welch's *t*-tests, *p*-values > 0.05).

These results suggest that overall CyclicLoess reduces variability most effectively and consistently than Median in both the 'corrupted' (TRC1) and good-quality (TRC2 and TRC3) data. Although Quantile and Qspline perform as well as CyclicLoess in the good-quality data, CyclicLoess outperforms them in the 'corrupted' data. Iset fails to improve over Median consistently.

### Signal in the normalized time course dataset

Since the aim of an effective normalization method is to remove noise while retaining biological signal in the data, we next compared the effectiveness of the normalization approaches in signal retention. As no spike-in datasets were available for CodeLink Bioarrays, we were unable to determine total signal in the data. Instead, we estimated signal by calculating the number of differentially expressed genes in the data. We expected that the more signal retained, the more differentially expressed genes should be revealed. Although similar methodology has been shown effective in our previous work [18], we developed a statistical model and used simulated data to illustrate the validity of this intuition (see additional file 1: Simulation model and data for detail).

**Figure 3:**
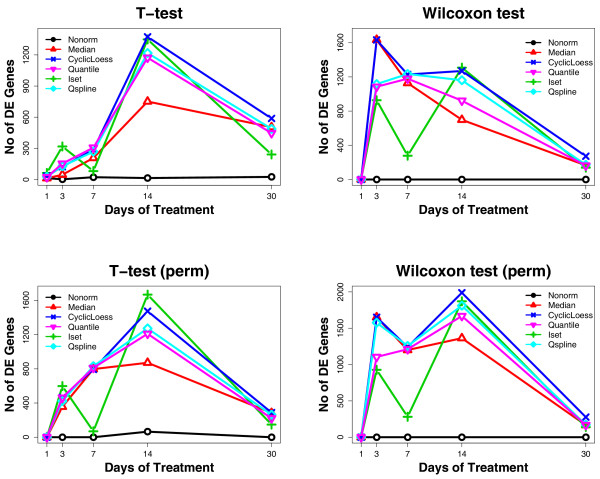
**Comparison of the numbers of differentially expressed genes estimated from the normalized time course dataset using multiple statistical tests**. The *x*-axis in each plot shows the days when the rats were treated. The *y*-axis represents the numbers of differentially expressed genes detected between the control vs. each test group using the following statistical tests: Welch's *t*-tests without permutation (T-test), Wilcoxon rank sum tests without permutation (Wilcoxon test), Welch's *t*-tests with permutation (T-test, perm), and Wilcoxon rank sum tests with permutation (Wilcoxon test, perm).

**Figure 4:**
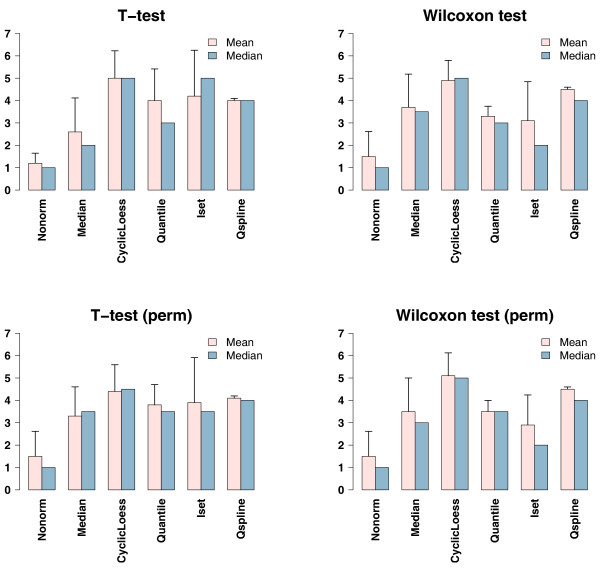
**Ranks of the normalization methods in the normalized time course dataset**. The mean, median and standard deviation of the ranks of the normalization method are defined in Table 1. The bar plots are visual representation of the results shown in Tables 1–4 (the "Mean Rank", "Median Rank" and "Standard Deviation" columns). In each plot, mean ranks are shown in pink, median ranks are in blue, and standard deviations of the ranks are shown as the error bars on top of the "Mean" rank bars.

We then used Welch's two sample *t*-tests and Wilcoxon rank sum tests, with and without permutation, to estimate the numbers of differentially expressed genes in the non-normalized data and in the data normalized with the five normalization methods. For each statistical test, Figure 3 shows the numbers of differentially expressed genes estimated from the normalized data at different time points. We ranked all the normalization methods at each time point based on the numbers of differentially expressed genes detected (Tables 1, 2, 3, 4). The more differentially expressed genes detected, the higher the rank of the normalization method. The mean, median and standard deviation of the ranks across all the time points were calculated for each normalization approach and are shown in Tables 1, 2, 3, 4 and Figure 4. For all the statistical tests performed, CyclicLoess has the highest mean and median ranks among all the normalization methods; the mean and median ranks of Qspline are also consistently higher than those of Median; Quantile fails to outperform Median in Wilcoxon rank sum tests (without permutation); Iset fails to improve over Median in Wilcoxon rank sum tests (both with and without permutation).

Notably, compared to the other normalization methods, Iset has the highest standard deviations of the ranks across all time points in almost all the statistical tests (3 out of the 4 tests) (Tables 1, 2, 3 and Figure 4). For example, the ranks of Iset range from 2 (in 7-day and 30-day treatments) to 6 (in 1-day and 3-day treatments) in Welch's *t*-tests without permutation (Table 1). It has been shown previously that the performance of Iset is dependent on the choice of the baseline array [17]; our results here suggest that even with a fixed choice of the baseline array, its performance may also depend on the arrays to be normalized.

In addition to the comparisons between the control group and each test group, we used the Analysis of Variance (ANOVA) to estimate the number of differentially expressed genes whose intensity values varied with the days of the treatment. CyclicLoess reveals the largest number of differentially expressed genes (68 genes) with ANOVA. Iset, Qspline and Quantile reveal slightly fewer numbers of differentially expressed genes (66, 56 and 54 genes, respectively), but they still significantly outperform Median and Nonorm (which reveal only 24 and 9 differentially expressed genes, respectively).

The comparison of the normalization methods in the time course dataset can be summarized as follows. For noise removal and signal retention, CyclicLoess demonstrates the greatest and most consistent improvement over Median; Qspline exhibits consistent yet moderate improvement over Median. Quantile performs consistently better than Median for variability reduction, yet does not do so for signal detection. Iset fails to improve over Median consistently for either noise reduction or signal retention.

Since CyclicLoess, Quantile and Qspline exhibit considerable improvement over Median in the time course dataset, we compared them in greater detail using another dataset, the IPF dataset (see Methods), which contains larger and more balanced numbers of arrays for both the control (control patients) and test groups (pulmonary fibrotic patients). To focus the comparison on these methods, we excluded Nonorm and Iset from further analyses.

### Variability of the normalized IPF dataset

Since there were no technical replicates in the IPF dataset, we compared the four normalization methods (CyclicLoess, Quantile, Qspline, and Median) for noise removal using the positive control probes on the CodeLink Bioarrays. The CV of the normalized intensity values across all arrays was calculated for each positive control probe in the IPF dataset processed with the normalization methods. Our results show that CyclicLoess, Quantile and Qspline all have significantly lower mean CVs (79%, 76% and 74%, respectively) than Median (89%). CyclicLoess has a slightly higher, yet not statistically significant mean CV than Quantile and Qspline (Welch's *t*-tests, *p*-values > 0.05).

### Signal in the normalized IPF dataset

**Figure 5:**
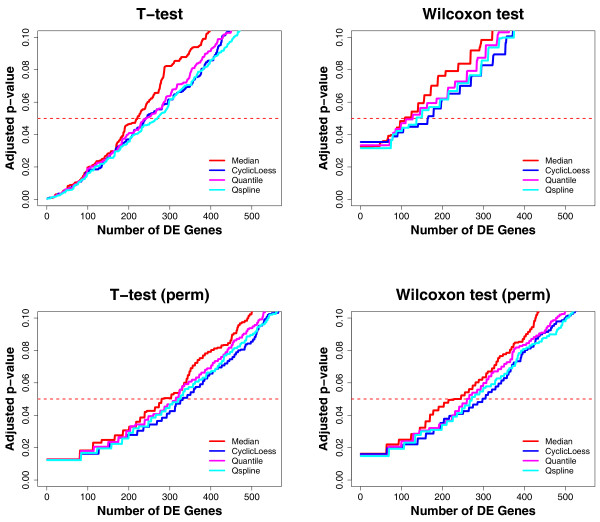
**Comparison of the numbers of differentially expressed genes estimated from the normalized IPF dataset using multiple statistical tests**. The *x*-axis shows the numbers of differentially expressed genes estimated using the following statistical tests: Welch's *t*-tests without permutation (T-test), Wilcoxon rank sum tests without permutation (Wilcoxon test), Welch's *t*-tests with permutation (T-test, perm), and Wilcoxon rank sum tests with permutation (Wilcoxon test, perm). The *y*-axis represents the *p*-values adjusted using the Benjamini & Hochberg FDR procedure. The red line shows the cut off adjusted *p*-value of 0.05.

Table 5 and Figure 5 show the numbers of differentially expressed genes estimated from the normalized IPF dataset using Welch's *t*-tests and Wilcoxon rank sum tests, with and without permutation. In three of the four statistical tests (*t*-tests with permutation and Wilcoxon tests with and without permutation, Figure 5), CyclicLoess reveals the largest numbers of differentially expressed genes (164, 331 and 297 genes, respectively) (Table 5), which are significantly greater than the numbers of differentially expressed genes revealed by Median (108, 279 and 227 genes, respectively). In all the statistical tests, Qspline reveals more differentially expressed genes than Quantile, which in turn outperforms Median (Table 5).

Overall, comparative results of the four normalization methods in the IPF dataset agree with most of those from the time course dataset: CyclicLoess and Qspline exhibit significant and consistent improvement over Median in both noise reduction and signal retention; CyclicLoess reveals slightly more signal (differentially expressed genes) than Qspline. Quantile outperforms Median for both noise reduction and signal retention in all four statistical tests, which is in contrast to its performance in the time course dataset where it fails to reveal more signal than Median in some tests (*e.g*., Wilcoxon rank sum tests, without permutation).

## Discussion

CodeLink Bioarrays are recently introduced, single-color oligonucleotide microarrays, which differ from Affymetrix GeneChips in the following aspects [22,26]: 1) CodeLink Bioarrays use a single pre-synthesized, pre-validated 30-mer probe to detect each target transcript, whereas GeneChips use multiple *in-situ *synthesized, 25-mer probes; and 2) the surface of CodeLink Bioarrays is made of 3-dimensional aqueous gel matrix, whereas that of Affymetrix GeneChips is made of 2-dimensional glass matrix. These characteristics suggest that CodeLink Bioarrays behave differently from GeneChips and may require different normalization strategies from the ones optimized for GeneChips.

In this study, in order to determine the best normalization method(s) for CodeLink Bioarrays, we compared five existing approaches designed for high-density oligonucleotide microarrays. These methods have been applied previously to Affymetrix GeneChip data. Our goal is to provide a guideline for practitioners in the choice of a 'proper' normalization method that removes variability and retains signal effectively for CodeLink Bioarray data and thus to ensure the validity of downstream data analyses. Using our criteria, the Median normalization method (recommended by the manufacturer) is insufficient for noise removal in the two examined CodeLink Bioarray datasets, whereas CyclicLoess and Qspline show considerable and consistent improvement over Median for both variability reduction and signal retention. CyclicLoess performs slightly better than Qspline for signal retention. Quantile exhibits moderate improvement over Median for variability reduction and signal retention in the IPF dataset, yet it fails to do so for signal retention in the time course dataset. Iset fails either to remove noise or to retain signal more effectively and consistently than Median in the time course dataset.

A major difference between CyclicLoess, Qspline, Quantile, and Iset can be explained as follows. CyclicLoess and Qspline have more relaxed assumptions on microarray data than Quantile and Iset. The former methods require only that genes on the arrays are randomly distributed (*i.e*., the numbers of up- and down-regulated genes are similar), whereas the latter approaches require either that the data on different arrays have the identical distribution (Quantile) or that few genes on the arrays are differentially expressed (Iset). Since we expected neither identical nor very different gene expression patterns between the control vs. test groups in the examined datasets, CyclicLoess and Qspline seem to be more reasonable choices for normalizing these data. This may account for their better performances in this work. Our results also partially agree with another comparative study using Affymetrix GeneChip data [27], which showed that CyclicLoess reduced variability and retained signal more effectively than Quantile. This suggests that CyclicLoess (and Qspline) may be more suitable than Quantile (and Iset) for normalizing single-color, oligonucleotide microarray data.

Besides the difference mentioned above, two baseline-array approaches, Qspline and Iset, also differ in the following way. Although both methods use a subset of genes to estimate intensity-dependent differences between a pair of microarrays for normalization, Qspline chooses these genes evenly over the entire range of the genes on the arrays. Iset, however, uses rank invariant genes, which are usually in small numbers (about 300 – 1000 genes in the examined datasets) and thus may be insufficient for estimating intensity effect accurately in some arrays. This may account for the unstable performance of Iset in the time course dataset. Moreover, although intuitively normalization should be more effective if a 'proper' set of 'housekeeping' genes can be selected, the effectiveness of such approach could be limited by the still unanswered question as to whether these genes exists in higher organisms [28].

Since Qspline is a baseline-array approach, a concern could be raised that its performance may depend on the choice of the baseline array. Indeed, it has been shown that the performance of Iset varied when different individual arrays were used as the baseline array [17]. However, when the 'synthetic' baseline array was employed such that the intensity value of each gene in this array is equal to the median or mean of the intensity values of the same gene across all arrays, the performance of Iset seemed to be stable compared to Nonorm and Quantile Normalization [17]. This suggests that these 'synthetic' baseline arrays (the ones we used in this study for Iset and Qspline) are less biased and therefore are better choices for baseline-array approaches.

In addition to the examined normalization methods, there are other approaches that can be applied to CodeLink Bioarray data. For example, mean cyclic loess [29] and fast linear loess [27] were designed as speed-up versions of CyclicLoess (which, as has been often noted, has the drawback of being computationally slow). Although proposed independently, mean cyclic loess and fast linear loess are virtually identical methods. Unlike CyclicLoess, which adjusts intensity-dependent differences between each pair of arrays by iterating over all pairs of arrays in the dataset (see Methods), the speed-up methods eliminate the iteration step. Instead, for each target array, they first make a reference array by having the intensity value of each gene equal to the mean intensity value of the same gene across the rest of the arrays, and then estimate and adjust intensity-dependent differences in the target array with respect to the reference array. These methods are similar to Qspline when the latter chooses a similar baseline array (*i.e*., using the array-wise mean intensity value for each gene to construct the baseline array, the one we used in this study).

There are two possible limitations in this study. The first is that in the time course dataset, the sample sizes of the control vs. test groups were not well balanced (*i.e*., the control group contained 14 arrays, while the test groups contained only 4 – 6 arrays each). Since a single statistical test could be biased or less powerful for estimating differentially expressed genes in unbalanced datasets, we used multiple statistical tests (parametric and non-parametric in nature, with and without permutation) to assess the performances of the normalization strategies. The conclusion of the comparative performances of the normalization methods was made based on the results from all the statistical tests. Moreover, we confirmed the evaluation results from the time course dataset using the IPF dataset. The latter dataset was better balanced in terms of the sample sizes of the control vs. test groups (11 and 15 arrays, respectively), and therefore allowed more accurate estimation of the numbers of the differentially expressed genes in the normalized data.

The second possible limitation of this study is that, since no spike-in datasets were available for CodeLink Bioarrays, two real CodeLink Bioarray datasets were used instead. It would be more informative if a fixed number of known differentially expressed genes were present in the data. However, this information is often unknown for real microarray datasets. Although spike-in or dilution data has been shown to be useful for evaluation of normalization methods [17,30], they are too clean in terms of the 'background' genes, whose intensity values are constant between control vs. test groups. In real data, these 'background' genes rarely exist. In fact, in the two examined datasets, we neither knew the number of 'background' genes in the control vs. test groups, nor did we even know the number of these genes within each group (since individual samples of each group were different). For this reason, normalization strategies that perform well in spike-in or dilution data may not perform equally well in real data. It may be the most informative if normalization methods can be evaluated using both real and spike-in microarray data.

## Methods

### Datasets

Two real datasets were used in this study.

#### Time course dataset

These data were collected to test the difference between a control group of rats (exposed to a treatment for 0 days) and test groups of rats exposed to a treatment for either 1, 3, 7, 14 or 30 days. The control group contained 14 arrays. The 14-day treatment group contained 6 arrays, and the other test groups contained 4 arrays each. Thus, the dataset contained 36 arrays. For the control group, there were 3 sets of technical replicates, sets TRC1, TRC2 and TRC3, which contained 5, 5 and 4 arrays, respectively. The arrays used in this dataset were CodeLink UniSet Rat I Bioarrays containing pre-validated oligonucleotide probes targeting about 10K transcripts in the rat genome.

#### IPF dataset

This dataset was generated to compare expression profiles of control lungs vs. lungs from patients with idiopathic pulmonary fibrosis (IPF). A total of 26 microarrays were obtained from 11 controls and 15 patients. The arrays used were CodeLink UniSet Human I Bioarrays containing pre-validated oligonucleotide probes targeting about 10K transcripts in the human genome. The arrays are available at the Gene Expression Omnibus (GEO accession number GSE 2052).

Both of the above CodeLink Bioarray platforms contain 68 bacterial control probes on each array, of which 18 are positive control probes (which can be used to monitor the quality of microarray experiments, see below) and 50 are negative control probes (which can be used to determine the low limit of signal). Each control probe is spotted 6 times on each array.

### Microarray protocol

A CodeLink Bioarray experiment involves the following steps. Total RNAs are first prepared from a biological sample. Then a set of bacterial mRNAs of known concentrations (which are provided by the manufacturers and have complementary sequences to the positive control probes on the Bioarrays) are spiked in as positive controls. The mixed mRNAs are reverse transcribed into cDNAs and amplified into cRNAs, using *in vitro *transcription. The cRNAs are labeled with a fluorescent dye and hybridized to a CodeLink Bioarray presynthesized with probes. Finally the array is washed and scanned. The intensity value (signal) of the fluorescent dye detected from each probe on the array is proportional to the abundance of the targeting transcript in the sample of interest. The quality of these processes can be monitored by detecting the signal of the positive control probes on the Bioarrays.

Since some normalization methods (*e.g*., Quantile and Qspline) do not perform well with missing values in microarray data, missing values in the raw data were imputed before normalization using the following procedure. For each dataset, we first removed genes which contained missing values in more than 5% of the microarrays, then used the *k*-NNimpute algorithm [31] to fill in missing values for the remaining genes. The *k*-NNimpute algorithm was implemented using the Bioconductor package/function **pamr/pamr.knnimpute(k = 10) **[32].

### Normalization methods

All of the examined normalization methods are available at [33].

#### Global approaches

We compared three global normalization methods, Median, CyclicLoess and Quantile. For reference, we also included Nonorm, which does not perform any data transformation on raw intensity values, *S*, of the spots on an array, which is defined as *S *= *S*_*f *_- *S*_*b*_, where *S*_*f *_is the fluorescent signal of the spots and *S*_*b *_is the local background intensity values of the spots. Median normalization scales the raw intensity values on an array using the median of the raw intensity value, *i.e*., *S*_*n *_= *S*/*median*(*S*), where *S*_*n *_is the normalized intensity values of the spots. Median is recommended by the manufacturer for normalization and serves as a baseline method in this study (Note: this should not be confused with the 'baseline-array' approaches described below). The Median-normalized CodeLink Bioarray data was obtained directly from the software provided by the manufacturer.

CyclicLoess [17] adjusts intensity-dependent differences between pairs of arrays with the aid of the MA plot (which, when used on data obtained from single-color microarrays, is the scatter plot of average log intensity values [*A*] of a pair of arrays vs. log intensity ratio [*M*] of the same arrays) [17]. CyclicLoess is an inter-microarray variant of loess-based normalization methods originally applied to two-color cDNA microarrays to adjust the intensity-dependent dye effect within a microarray [19,24]. In two-color cDNA microarrays, a test- and a reference sample (labeled with two fluorescent dyes of distinct colors, *i.e*., red and green, respectively) are hybridized to the same array; the differences of gene expression between these samples can be obtained by comparing the red and green fluorescent intensities of each gene on the same array. Thus intra-microarray loess-based normalization approaches can be used to adjust intensity-dependent differences in each microarray. In single-color microarrays, however, only one sample is hybridized to each microarray, and the differences of gene expression between different samples can only be obtained by comparing intensity values of each gene on different arrays. Inter-microarray normalization methods (*e.g*., CyclicLoess studied here), therefore, have to be used to estimate and minimize intensity-dependent differences in these microarrays.

CyclicLoess uses the MA plot and loess smoothing [34] to estimate intensity-dependent differences in a pair of microarrays, and then removes these differences by centering the loess line to zero. This procedure is carried out in a pairwise manner for all the arrays in a dataset and iterated until intensity-dependent differences are removed from all arrays. CyclicLoess was implemented using the Bioconductor package/function **affy/normalize.loess(data, epsilon = 1, log.it = F, span = 0.4, maxit = 2) **[35]. The parameter epsilon in **normalize.loess **measures intensity-dependent differences in the data and thus serves as a criterion for the procedure to stop iterating. Our results show that when epsilon is smaller than 1, intensity-dependent differences in the data are negligible. The smaller value of epsilon does not produce better results in terms of variability reduction and signal retention (data not shown). It took CyclicLoess two iterations in the time course dataset and one iteration in the IPF dataset to satisfy the stopping criterion (epsilon < 1).

Quantile [17] normalizes data in the following way. It first ranks data on each array, and then assigns data of the same rank across all arrays the mean of the data (of the same rank). Quantile normalization was implemented using the Bioconductor package/function **affy/normalize.quantiles(data)**.

#### Baseline-array approaches

We compared two baseline-array methods, Iset and Qspline. These two strategies share several similarities: 1) both need to choose a baseline-array to estimate intensity-dependent differences in target arrays; 2) both use a spline smoothing technique to remove intensity-dependent differences in target arrays; 3) both estimate smoothing curves for normalization using a subset of the genes on the arrays; and 4) both are rank-based methods. However, they differ in their choices of the subset of genes for fitting normalization curves. Iset chooses these genes by selecting a set of rank-invariant genes (or so called "housekeeping genes") in the target array with respect to the baseline array [36,37], while Qspline does so by first ranking all the genes on the target array and the baseline array respectively, and then by using quantiles of the ranked genes to estimate smoothing curves [38].

Iset was implemented in the Bioconductor package/function **affy/normalize.invariantset ("target", "ref", prd.td = c(0.003, 0.007))**, where "target" is the data matrix from the target array and "ref" is the data matrix from the baseline array. We chose the baseline array for Iset as follows: the intensity value of each gene is the median of the intensity values of the same gene across all arrays. Qspline was implemented in the Bioconductor package/function **affy/normalize.qspline (data, fit.iters = 5, min.offset = 5, spar = 0, p.min = 0, p.max = 1.0)**. We chose the baseline array for Qspline using the default option, in which each gene in the baseline array had the intensity value equal to the mean of the intensity values of the same gene across all arrays.

### Detection of noise in the normalized datasets

We applied the five normalization methods individually to the time course and IPF datasets. In order to assess the effectiveness of the normalization methods in removing noise, we first used three sets of technical replicate microarrays, sets TRC1, TRC2 and TRC3, from the time course dataset. For each set of the replicates, we used pairwise MA plots to examine intensity-dependent differences in the data that are normalized individually with the normalization methods; we then calculated the coefficient of variation (CV) of the normalized intensity values for each transcript (gene) across all arrays. Specifically, if we let *S*_*k *_denote the vector of normalized intensity values for transcript *k *in technical replicate set *j*, *S*_*k *_= (*s*_*k*,1_,..., *s*_*k*,*m*_,...*s*_*k*,*M*_) where *m *denotes the *m*th array in set *j *and 1 ≤ *m *≤ *M*. The CV of *S*_*k *_is computed as follows:

CV (*S*_*k*_) = standard deviation (*S*_*k*_)/mean (*S*_*k*_) × 100%.

Finally, we exploited the redundancy in the positive control probes on the arrays and measured the CVs for these probes across all arrays in the time course dataset. Similarly as above, we let *S*_*c *_denote the vector of normalized intensity values for the positive control probe *c *on array *n*, and *S*_*c *_= (*s*_*c*,1,1_,...*s*_*c*,*p*,1_,...*s*_*c*,6,1_,...,*s*_*c*,*p*,*n*_,...*s*_*c*,6,*N*_), where *p *denotes the *p*th positive control probe *c *on array *n*, 1 ≤ *p *≤ 6 (*i.e*., each positive control probe is spotted six times on each array), where 1 ≤ *n *≤ *N *and *N *(= 36) is the total number of arrays in the entire time course datasets. The CV of *S*_*c *_is computed as follows:

CV (*S*_*c*_) = standard deviation (*S*_*c*_)/mean (*S*_*c*_) × 100%.

Since the IPF dataset did not contain technical replicates, we measured and compared the CVs for each positive control probe across all arrays in the IPF data normalized with the normalization methods. The normalized intensity values used in this section for calculating CVs are on the scale of the raw intensity data (*i.e*., not log-transformed values).

### Detection of signal in the normalized datasets

A negative control threshold *T*_*NC *_is used (as suggested by CodeLink Bioarrays) to monitor the low limit of signal [39], where *T*_*NC *_is defined as *T*_*NC *_= (80% trimmed mean of negative control probes) + (3 standard deviations of the 80% trimmed population of negative control probes). In order to minimize the effects of low signal probes (whose intensity values are smaller than the negative control threshold) on signal detection, we replaced intensity values of these probes with *T*_*NC*_.

Log-transformed, normalized intensity values were used in the analyses described below.

In order to compare the effectiveness of the normalization methods in enhancing signal reproducibility, we detected signal in the normalized datasets. We assume that signal quality can be estimated by the number of differentially expressed genes detected (that is, the more signal retained in the normalized data, the more differentially expressed genes should be revealed). We first developed a simulation model and verified this intuition using simulated data (see additional file1: Simulation model and data for detail).

We then used multiple statistical significance tests, both parametric (*e.g*., Welch's two-sample *t*-tests and ANOVA) and non-parametric (*e.g*., Wilcoxon rank sum tests), to detect differentially expressed genes in the normalized datasets. Raw *p*-values of the *t*-statistics and Wilcoxon rank sum tests were computed using the null distribution of the test statistics as well as the permutation tests of the sample labels. These *p*-values were then adjusted for multiple testing using the false discovery rate (FDR) controlling procedure of Benjamini & Hochberg [40], which seems to balance the control of both false-positive and false-negative error rates better than other procedures (*e.g*., Bonferroni, the Benjamini & Yekutieli FDR and SAM) [41]. The cut-off adjusted *p*-value of 0.05 was used to determine differentially expressed genes for these two-sample statistical tests.

In addition to pair-wise comparisons between the control and test groups, we used ANOVA to detect genes whose intensity values varied with the length of the treatment. Since we believe that there was a non-linear relationship between the response- (intensity values of genes) and the explanatory variables (days of the treatment), we used a quadratic regression model to fit these variables. For transcript *k*, where 1 ≤ *k *≤ *K *and *K *is the total number of the transcripts on the array, let *j *be the treatment group, 1 ≤ *j *≤ 6; for array *i *in treatment group *j *and 1 ≤ *i *≤ *N*_*j*_, where *N*_*j *_is the number of the arrays for treatment group *j*, let *s*_*k*,*i*,*j *_denote the intensity value of transcript *k *on array *i *in treatment group *j *and *d*_*i*,*j *_be the day of treatment for array *i *in treatment group *j*, *i.e*., *d*_*i*,*j *_= {0,1,3,7,14,30}. The quadratic regression model can be written as:

sk,i,j=β0+β1di,j+β2di,j2+εk,i,j,εk,i,j~N(0,σ2).
 MathType@MTEF@5@5@+=feaafiart1ev1aaatCvAUfKttLearuWrP9MDH5MBPbIqV92AaeXatLxBI9gBaebbnrfifHhDYfgasaacH8akY=wiFfYdH8Gipec8Eeeu0xXdbba9frFj0=OqFfea0dXdd9vqai=hGuQ8kuc9pgc9s8qqaq=dirpe0xb9q8qiLsFr0=vr0=vr0dc8meaabaqaciGacaGaaeqabaqabeGadaaakeaafaqabeGabaaabaGaem4Cam3aaSbaaSqaaiabdUgaRjabcYcaSiabdMgaPjabcYcaSiabdQgaQbqabaGccqGH9aqpcqaHYoGydaWgaaWcbaGaeGimaadabeaakiabgUcaRiabek7aInaaBaaaleaacqaIXaqmaeqaaOGaemizaq2aaSbaaSqaaiabdMgaPjabcYcaSiabdQgaQbqabaGccqGHRaWkcqaHYoGydaWgaaWcbaGaeGOmaidabeaakiabdsgaKnaaDaaaleaacqWGPbqAcqGGSaalcqWGQbGAaeaacqaIYaGmaaGccqGHRaWkcqaH1oqzdaWgaaWcbaGaem4AaSMaeiilaWIaemyAaKMaeiilaWIaemOAaOgabeaakiabcYcaSaqaaiabew7aLnaaBaaaleaacqWGRbWAcqGGSaalcqWGPbqAcqGGSaalcqWGQbGAaeqaaOGaeiOFa4NaemOta40aaeWaaeaacqaIWaamcqGGSaalcqaHdpWCdaahaaWcbeqaaiabikdaYaaaaOGaayjkaiaawMcaaiabc6caUaaaaaa@6581@

ANOVA was used to estimate statistical significance of the model parameters *β*_1 _and *β*_2 _for all transcripts, 1 ≤ *k *≤ *K*. The *p*-values of the F statistics were adjusted using the Benjamini & Hochberg FDR procedure. To ensure that both *β*_1 _and *β*_2 _are non-zero and thus minimize the risk of false positives, a stringent criterion, the adjusted *p*-value < 0.01, was used to determine differentially expressed genes.

## List of abbreviations

ANOVA: Analysis of Variance;

CV: coefficient of variation;

FDR: false discovery rate;

IPF: idiopathic pulmonary fibrosis;

loess: local regression estimation.

## Authors' contributions

WW designed and performed computational experiments, and drafted the manuscript. ND performed microarray experiments. GCT conducted the simulation study and edited the manuscript. TR read and edited the manuscript. EPX and NK participated in experimental design and in drafting the manuscript. All authors contributed to, read and approved the final manuscript.

## Supplementary Material

Additional File 1 Simulation model and data Click here for file

## Figures and Tables

**Table 1:** Ranks of the normalization methods in Welch's *t*-tests (without permutation) in the time course dataset. For each time point, the normalization strategies were ranked based on the numbers of differentially expressed genes estimated from the normalized data using Welch's *t*-tests without permutation. The more differentially expressed genes detected, the higher the rank of the normalization method. The mean, median and standard deviation of the ranks across all the time points were calculated for each method and are shown in the "Mean Rank", "Median Rank" and "Standard Deviation" columns, respectively. For each column in the table, the highest mean, median and standard deviation of the ranks are shown in bold.

Normalization Method	Rank					Mean Rank	Median Rank	Standard Deviation
				
	1d	3d	7d	14d	30d			
Nonorm	2	1	1	1	1	1.2	1	0.4
Median	1	2	3	2	5	2.6	2	1.5
CyclicLoess	5	3	5	**6**	**6**	**5.0**	**5**	1.2
Quantile	3	5	**6**	3	3	4.0	3	1.4
Iset	**6**	**6**	2	5	2	4.2	5	**2.0**
Qspline	4	4	4	4	4	4.0	4	0

**Table 2:** Ranks of the normalization methods in Wilcoxon tests (without permutation) in the time course dataset. For each time point, the normalization strategies were ranked based on the numbers of differentially expressed genes estimated from the normalized data using Wilcoxon rank sum tests without permutation. "Rank" and the "Mean", "Median" and "Standard Deviation" of the ranks are defined as described in Table 1. For each column in the table, the highest rank(s), the mean, median and standard deviation of the ranks are shown in bold.

Normalization Method	Rank					Mean Rank	Median Rank	Standard Deviation
				
	1d	3d	7d	14d	30d			
Nonorm	**3.5**	1	1	1	1	1.5	1	1.1
Median	**3.5**	**6**	3	2	4	3.7	3.5	1.5
CyclicLoess	**3.5**	5	5	5	**6**	**4.9**	**5**	0.9
Quantile	**3.5**	3	4	3	3	3.3	3	0.4
Iset	**3.5**	2	2	**6**	2	3.1	2	**1.7**
Qspline	3.5	4	6	4	5	4.5	4	1

**Table 3:** Ranks of the normalization methods in Welch's *t*-tests (with permutation) in the time course dataset. For each time point, the normalization strategies were ranked based on the numbers of differentially expressed genes estimated from the normalized data using Welch's *t*-tests with permutation. "Rank" and the "Mean", "Median" and "Standard Deviation" of the ranks are defined as described in Table 1. For each column in the table, the highest rank(s), the mean, median and standard deviation of the ranks are shown in bold.

Normalization Method	Rank					Mean Rank	Median Rank	Standard Deviation
				
	1d	3d	7d	14d	30d			
Nonorm	**3.5**	1	1	1	1	1.5	1	1.1
Median	**3.5**	2	4	2	5	3.3	3.5	1.3
CyclicLoess	**3.5**	4.5	3	5	**6**	**4.4**	**4.5**	1.2
Quantile	**3.5**	4.5	5	3	3	3.8	3.5	0.9
Iset	**3.5**	**6**	2	**6**	2	3.9	3.5	**2.0**
Qspline	3.5	3	6	4	4	4.1	4	1.1

**Table 4:** Ranks of the normalization methods in Wilcoxon tests (with permutation) in the time course dataset. For each time point, the normalization strategies were ranked based on the numbers of differentially expressed genes estimated from the normalized data using Wilcoxon rank sum tests with permutation. "Rank" and the "Mean", "Median" and "Standard Deviation" of the ranks are defined as described in Table 1. For each column in the table, the highest rank(s), the mean, median and standard deviation of the ranks are shown in bold.

Normalization Method	Rank					Mean Rank	Median Rank	Standard Deviation
				
	1d	3d	7d	14d	30d			
Nonorm	**3.5**	1	1	1	1	1.5	1	1.1
Median	**3.5**	**6**	3	2	3	3.5	3	**1.5**
CyclicLoess	**3.5**	5	5	**6**	**6**	**5.1**	**5**	1.0
Quantile	**3.5**	3	4	3	4	3.5	3.5	0.5
Iset	**3.5**	2	2	5	2	2.9	2	1.3
Qspline	**3.5**	4	**6**	4	5	4.5	4	1.0

**Table 5:** Numbers of differentially expressed genes estimated from the IPF dataset. Columns show the normalization methods used to process the IPF dataset. Rows show the statistical tests performed to detect the numbers of differentially expressed genes (adjusted *p*-values < 0.05) from the normalized data. For each statistical test, the largest number of differentially expressed genes is shown in bold.

	Median	CyclicLoess	Quantile	Qspline
Welch's *t*-test	220	240	242	**269**
Wilcoxon test	108	**164**	127	136
Welch's *t*-test (perm)	279	**331**	314	319
Wilcoxon test (perm)	227	**297**	259	271

## References

[B1] Eisen MB, Spellman PT, Brown PO, Botstein D (1998). Cluster analysis and display of genome-wide expression patterns. Proc Natl Acad Sci U S A.

[B2] Brown MP, Grundy WN, Lin D, Cristianini N, Sugnet CW, Furey TS, Ares MJ, Haussler D (2000). Knowledge-based analysis of microarray gene expression data by using support vector machines. Proc Natl Acad Sci U S A.

[B3] Kaminski N, Allard JD, Pittet JF, Zuo F, Griffiths MJ, Morris D, Huang X, Sheppard D, Heller RA (2000). Global analysis of gene expression in pulmonary fibrosis reveals distinct programs regulating lung inflammation and fibrosis. Proc Natl Acad Sci U S A.

[B4] Golub TR, Slonim DK, Tamayo P, Huard C, Gaasenbeek M, Mesirov JP, Coller H, Loh ML, Downing JR, Caligiuri MA, Bloomfield CD, Lander ES (1999). Molecular classification of cancer: class discovery and class prediction by gene expression monitoring. Science.

[B5] Alizadeh AA, Eisen MB, Davis RE, Ma C, Lossos IS, Rosenwald A, Boldrick JC, Sabet H, Tran T, Yu X, Powell JI, Yang L, Marti GE, Moore T, Hudson JJ, Lu L, Lewis DB, Tibshirani R, Sherlock G, Chan WC, Greiner TC, Weisenburger DD, Armitage JO, Warnke R, Levy R, Wilson W, Grever MR, Byrd JC, Botstein D, Brown PO, Staudt LM (2000). Distinct types of diffuse large B-cell lymphoma identified by gene expression profiling. Nature.

[B6] Bhattacharjee A, Richards WG, Staunton J, Li C, Monti S, Vasa P, Ladd C, Beheshti J, Bueno R, Gillette M, Loda M, Weber G, Mark EJ, Lander ES, Wong W, Johnson BE, Golub TR, Sugarbaker DJ, Meyerson M (2001). Classification of human lung carcinomas by mRNA expression profiling reveals distinct adenocarcinoma subclasses. Proc Natl Acad Sci U S A.

[B7] Clark EA, Golub TR, Lander ES, Hynes RO (2000). Genomic analysis of metastasis reveals an essential role for RhoC. Nature.

[B8] Segal E, Friedman N, Kaminski N, Regev A, Koller D (2005). From signatures to models: understanding cancer using microarrays. Nat Genet.

[B9] Pardo A, Gibson K, Cisneros J, Richards TJ, Yang Y, Becerril C, Yousem S, Herrera I, Ruiz V, Selman M, Kaminski N (2005). Up-regulation and profibrotic role of osteopontin in human idiopathic pulmonary fibrosis. PLoS Med.

[B10] Ning W, Li CJ, Kaminski N, Feghali-Bostwick CA, Alber SM, Di YP, Otterbein SL, Song R, Hayashi S, Zhou Z, Pinsky DJ, Watkins SC, Pilewski JM, Sciurba FC, Peters DG, Hogg JC, Choi AM (2004). Comprehensive gene expression profiles reveal pathways related to the pathogenesis of chronic obstructive pulmonary disease. Proc Natl Acad Sci U S A.

[B11] Davidson LA, Nguyen DV, Hokanson RM, Callaway ES, Isett RB, Turner ND, Dougherty ER, Wang N, Lupton JR, Carroll RJ, Chapkin RS (2004). Chemopreventive n-3 polyunsaturated fatty acids reprogram genetic signatures during colon cancer initiation and progression in the rat. Cancer Res.

[B12] Hu J, Gray CA, Spencer TE (2004). Gene expression profiling of neonatal mouse uterine development. Biol Reprod.

[B13] Peng Y, Kang Q, Cheng H, Li X, Sun MH, Jiang W, Luu HH, Park JY, Haydon RC, He TC (2003). Transcriptional characterization of bone morphogenetic proteins (BMPs)-mediated osteogenic signaling. J Cell Biochem.

[B14] Hartemink AJ, Gifford DK, Jaakkola TS, Young RA, Bittner ML, Chen Y, Dorsel AN and Dougherty ER (2001). Maximum-likelihood estimation of optimal scaling factors for expression array normalization: ; San Jose, California..

[B15] Park T, Yi SG, Kang SH, Lee S, Lee YS, Simon R (2003). Evaluation of normalization methods for microarray data. BMC Bioinformatics.

[B16] Wu W, Xing EP, Myers C, Mian IS, Bissell MJ (2005). Evaluation of Normalization Methods for cDNA Microarray Data by k-NN Classification. BMC Bioinformatics.

[B17] Bolstad BM, Irizarry RA, Astrand M, Speed TP (2003). A comparison of normalization methods for high density oligonucleotide array data based on variance and bias. Bioinformatics.

[B18] Barash Y, Dehan E, Krupsky M, Franklin W, Geraci M, Friedman N, Kaminski N (2004). Comparative analysis of algorithms for signal quantitation from oligonucleotide microarrays. Bioinformatics.

[B19] Yang YH, Dudoit S, Luu P, Speed TP, Bittner ML, Chen Y, Dorsel AN and Dougherty ER (2001). Normalization for cDNA microarray data: ; San Jose, California..

[B20] Cui X, Kerr MK, Churchill GA (2003). Transformations for cDNA Microarray Data. Statistical Applications in Genetics and Molecular Biology.

[B21] Tseng GC, Oh MK, Rohlin L, Liao JC, Wong WH (2001). Issues in cDNA microarray analysis: quality filtering, channel normalization, models of variations and assessment of gene effects. Nucleic Acids Res.

[B22] Shippy R, Sendera TJ, Lockner R, Palaniappan C, Kaysser-Kranich T, Watts G, Alsobrook J (2004). Performance evaluation of commercial short-oligonucleotide microarrays and the impact of noise in making cross-platform correlations. BMC Genomics.

[B23] Zien A, Aigner T, Zimmer R, Lengauer T (2001). Centralization: a new method for the normalization of gene expression data. Bioinformatics.

[B24] Dudoit S, Yang YH, Callow MJ, Speed TP (2000). Statistical methods for identifying differentially expressed genes in replicated cDNA microarray experiments..

[B25] Ihaka R, Gentleman R (1996). R: A Language for Data Analysis and Graphics. Journal of Computational and Graphical Statistics.

[B26] Amersham Biosciences: Human Bioarrays for Gene Expression. http://www4.amershambiosciences.com/aptrix/upp01077.nsf/Content/codelink_human_bioarrays.

[B27] Ballman KV, Grill DE, Oberg AL, Therneau TM (2004). Faster cyclic loess: normalizing RNA arrays via linear models. Bioinformatics.

[B28] Bissell MJ (1981). The differentiated state of normal and malignant cells or how to define a "normal" cell in culture. Int Rev Cytol.

[B29] Edwards D (2003). Non-linear normalization and background correction in one-channel cDNA microarray studies. Bioinformatics.

[B30] Cope LM, Irizarry RA, Jaffee HA, Wu Z, Speed TP (2004). A benchmark for Affymetrix GeneChip expression measures. Bioinformatics.

[B31] Troyanskaya O, Cantor M, Sherlock G, Brown P, Hastie T, Tibshirani R, Botstein D, Altman RB (2001). Missing value estimation methods for DNA microarrays. Bioinformatics.

[B32] Hastie T, Tibshirani R, Narasimhan B, Chu G (2003). Pam: prediction analysis for microarrays. http://cran.us.r-project.org/src/contrib/PACKAGES.html#pamr.

[B33] Gentleman RC, Carey VJ, Bates DM, Bolstad B, Dettling M, Dudoit S, Ellis B, Gautier L, Ge Y, Gentry J, Hornik K, Hothorn T, Huber W, Iacus S, Irizarry R, Leisch F, Li C, Maechler M, Rossini AJ, Sawitzki G, Smith C, Smyth G, Tierney L, Yang JY, Zhang J (2004). Bioconductor: open software development for computational biology and bioinformatics. Genome Biol.

[B34] Cleveland WS, Devlin SJ (1988). Locally Weighted Regression: An Approach to Regression Analysis by Local Fitting. Journal of the American Statistical Association.

[B35] Bolstad B (2005). affy: Built-in Processing Methods. http://www.bioconductor.org/.

[B36] Schadt EE, Li C, Ellis B, Wong WH (2001). Feature extraction and normalization algorithms for high-density oligonucleotide gene expression array data. J Cell Biochem Suppl.

[B37] Li C, Wong WH, Parmigiani G, Garrett ES, Irizarry RA and Zeger SL (2003). DNA-Chip Analyzer (dChip). The Analysis of Gene Expression Data: Methods and Software.

[B38] Workman C, Jensen LJ, Jarmer H, Berka R, Gautier L, Nielser HB, Saxild HH, Nielsen C, Brunak S, Knudsen S (2002). A new non-linear normalization method for reducing variability in DNA microarray experiments. Genome Biol.

[B39] Amersham Biosciences: Improved methodology for assessing the lower limit of detection. http://www4.amershambiosciences.com/APTRIX/upp00919.nsf/content/AE91888890AFC5F9C1256EC000084AD3?OpenDocument&hometitle=search.

[B40] Benjamini Y, Hochberg Y (1995). Controlling the false discovery rate: A practical and powerful approach to multiple testing. J Roy Statist Soc Ser B.

[B41] Dudoit S, Shaffer JP, Boldrick JC (2003). Multiple hypothesis testing in microarray experiments. Statistical Science.

