# Supplementary material for: Comparison of normalization methods for CodeLink Bioarray data
Source: BMC Bioinformatics. 2005 Dec 28;6:309. doi: 10.1186/1471-2105-6-309 (PMC1373657; doi:10.1186/1471-2105-6-309)
Supplement: Additional File 1 — Simulation model and data [file 1471-2105-6-309-S1.pdf]

SUPPORTING MATERIAL

SIMULATION MODEL AND DATA

The simulation model was developed to verify the intuition that signal quality can be estimated by the number of differentially expressed genes detected. Suppose we have arrays of n_1 control samples and n_2 test samples. Let x_{gi} and y_{gj} represent the resulting normalized intensity values in gene *g* of control sample *i* and test sample *j*, respectively and ($g = 1, \ldots, G$; $i = 1, \ldots, n_1$; $j = 1, \ldots, n_2$). Consider the following model:

$$x_{gi} = \mu_{g1} + \delta_{gi} + \varepsilon_{gi},$$

$$y_{gi} = \mu_{g2} + \delta_{gj} + \varepsilon_{gj}.$$

where μ_1 and μ_2 are the true expected intensity values of the control and test groups, respectively; δ represents the errors introduced by the normalization method, noise and other unknown errors; and ε represents biological variations. For simplicity, suppose δ and ε are independent and

$$\delta_{gi} \sim N(0, \sigma_\delta^2),$$

$$\varepsilon_{gi} \sim N(0, \sigma_\varepsilon^2).$$

We assume an effective normalization approach should introduce small random error in δ and thus small σ_δ.

In the simulation study, we supposed $G = 10000$, $n_1 = 11$ and $n_2 = 15$, which was the condition similar to that in the IPF dataset. We further assumed that the first 400 genes were differentially expressed (*i.e.*, total signal in the data) such that $\sigma_\varepsilon / |\mu_1 - \mu_2| = 0.5$ and for the remaining 9600 genes $\mu_1 - \mu_2 = 0$ and $\sigma_\varepsilon = 0.5$. Welch's two-sample *t*-tests were applied for testing differentially expressed genes and raw *p*-values were adjusted for multiple testing using the Benjamini & Hochberg FDR procedure.

The results in Figure s1 show that the smaller the σ_δ (and thus the smaller the random error present in the data), the more differentially expressed genes can indeed be revealed from the data.

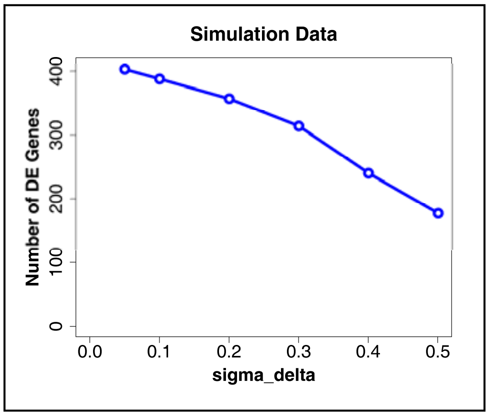

Figure s1: Numbers of differentially expressed genes estimated from simulated data with various degrees of random errors. The *x*-axis in the plot represents the variance of random errors σ_δ present in the data. The *y*-axis shows the numbers of differentially expressed genes detected from the data.
